# Effect of oliceridine versus morphine on the quality of early recovery after thoracoscopic surgery: a randomized, controlled clinical trial

**DOI:** 10.1186/s12871-026-03674-6

**Published:** 2026-02-13

**Authors:** Xueqing Na, Ying Chen, Xin Liu, Jian Yang, Miao Tan, Yun Zhou, Man Shi, Jie Ouyang, Yongyu Si

**Affiliations:** Department of Anesthesiology, The Second Affiliated Hospital of Kunming Medical University, Kunming, Yunnan Province 650033 China

**Keywords:** Early recovery quality, Thoracoscopic surgery, Oliceridine, Time to extubation

## Abstract

**Background:**

Thoracic surgery is frequently accompanied by severe pain. Although opioids remain the primary choice for effective analgesia in acute moderate-to-severe pain, their use is limited by adverse effects. Oliceridine, a novel G protein-biased μ-receptor agonist, has demonstrated a potentially improved safety compared to traditional opioid medications. Therefore, we investigated a short-course regimen of oliceridine for postoperative analgesia to evaluate its effects on early recovery quality in patients undergoing video-assisted thoracoscopic surgery (VATS).

**Methods:**

A total of 100 patients (ages 18-75 years) scheduled for elective thoracoscopic surgery were enrolled and randomly assigned to either the oliceridine group or the morphine group (n = 50 per group). Standardized anesthesia protocols were implemented. At the conclusion of surgery, patients received an intravenous loading dose of either oliceridine (1.5 mg) or morphine (4 mg). Supplemental analgesia (oliceridine 0.5 mg or morphine 1 mg) was administered as needed in the post-anesthesia care unit (PACU). The primary outcome measure was time to extubation. Secondary outcome measures included time to alertness recovery, Numerical Rating Scale (NRS) pain scores, requirement for analgesic rescue, adverse events during PACU stay, Quality of Recovery-15 (QoR-15) scores at 24 h postoperatively, time to first oral intake, time to first ambulation, and incidence of 24-h gastrointestinal adverse events.

**Results:**

A total of 95 patients completed the study (Oliceridine group: n = 46, Morphine group: n = 49). Compared with the Morphine group, the Oliceridine group demonstrated a shorter time to extubation (12 ± 4 min vs. 20 ± 8 min, mean difference -7.6 min,95% CI -10.4 – -4.9, *P* < 0.001); faster alertness recovery (18 ± 5 min vs. 29 ± 10 min, *P* < 0.001); and lower NRS pain scores at 5 min post-extubation (3 points vs. 5 points, *P* < 0.001); a lower proportion of patients requiring analgesic rescue (45.7% vs. 77.6%, *P* = 0.001); fewer overall adverse events occurred during PACU stay (19.6% vs. 38.8%, *P* = 0.04). However, no statistically significant differences were observed between the two groups in postoperative recovery metrics assessed at 24 h, including QoR-15 scores, time to first oral intake, time to first ambulation, and gastrointestinal adverse events (*P* > 0.05).

**Conclusion:**

Oliceridine demonstrated significant early recovery benefits compared to morphine in VATS patients, including faster extubation, quicker return of alertness, superior immediate analgesia, and fewer PACU adverse events. These advantages, however, were restricted to the immediate postoperative period and did not extend to 24-h recovery metrics. Therefore, while oliceridine is a potential option for optimizing early recovery pathways, its longer-term efficacy and role require further investigation.

**Trial registration:**

Randomized controlled trial; Chinese Clinical Trial Registration, ChiCTR2500096716 (Date 05/02/2025).

This study was reported in accordance with the CONSORT guidelines (Consolidated Standards of Reporting Trials).

## Introduction

Enhanced Recovery After Surgery (ERAS) represents a prominent research focus in surgical fields. It is a multidisciplinary, multimodal clinical improvement pathway designed to maximize patient recovery after surgery. In 2019, the Enhanced Recovery After Thoracic Surgery (ERATS) protocol was initially published by the ERAS® Society and the European Society of Thoracic Surgeons [1], and has been continuously updated [2–4]. ERATS implementation has been demonstrated to reduce healthcare costs and improve patient outcomes [5].

Thoracic surgery is frequently accompanied by intense postoperative pain. Lack of adequate management of postoperative pain can have a significant impact on patients’ outcome, including delayed recovery time, extended hospitalization and higher health-care costs. Therefore, postoperative pain management constitutes an important element within ERATS protocols for facilitating patient recovery [1]. Traditional opioid analgesics remain first-line agents for managing moderate-to-severe acute pain as classic potent analgesics, despite their clinical application has been limited due to dose-related adverse events. Oliceridine, a novel μ-opioid receptor agonist approved in both the United States and China, (but not currently by the European Medicines Agency), has demonstrated significantly fewer adverse effects than conventional opioid medications owing to reduced β-arrestin recruitment. Two Phase III studies have confirmed the efficacy and safety of oliceridine as an analgesic for postoperative pain management in abdominal and orthopedic surgeries [6, 7], but whether the administration of oliceridine would benefit patients having thoracoscopic surgery is unclear. Therefore, we conducted this randomized controlled trial to investigate its effects on patients' early recovery quality.

## Methods

### Ethics approval and trial registration

This prospective, randomized, double‑blind, controlled trial was conducted in accordance with the Declaration of Helsinki and approved by the Medical Ethics Committee of the Second Affiliated Hospital of Kunming Medical University. The original protocol was approved under No. PJ-Ke-2024-292. Prior to study initiation, the protocol was revised regarding the randomization scheme, blinding procedure, morphine loading dose, and secondary outcomes. All revisions were finalized and approved before patient enrollment (protocol amendment approval date: 10 February 2025; amended protocol approval No.: YJ-2025-373; first patient enrolled: 17 February 2025). Written informed consent was obtained from all participants. The trial was prospectively registered in the Chinese Clinical Trial Registry (ChiCTR) on 5 February 2025 (registration identifier: ChiCTR2500096716), and all protocol amendments have been updated in the registry.

### Randomization and blinding

Randomization was performed on the day of surgery after the patient's arrival in the operating room but before the induction of anesthesia. Patients were allocated in a 1:1 ratio to one of the two groups using computer-generated block randomization sequences (block size of 4–6). The allocation results were concealed in sequentially numbered, opaque, sealed envelopes, which were managed by research nurses not involved in outcome assessment. These research nurses prepared and administered all postoperative analgesics. Both the patients and the attending anesthesiologists were blinded to the group assignments.

### Participants

We enrolled patients aged 18–75 years, with ASA physical status I-III and a body mass index (BMI) of 18.5–30 kg/m^2^, scheduled for thoracoscopic surgery under general anesthesia, including procedures such as lobectomy and mediastinal tumor resection. Exclusion criteria were: preoperative respiratory failure; advanced cancer with extensive metastasis or requiring other adjuvant therapies; diagnosis of sleep apnea syndrome; hepatic or renal insufficiency; long-term use of sedatives; prolonged corticosteroid therapy; psychiatric disorders; lactation period; preoperative chronic pain (persisting > 3 months); and hypersensitivity to the investigational drugs.

### Interventions

Patients were randomized to the Oliceridine group or Morphine group according to the interventions, receiving oliceridine or morphine respectively for postoperative analgesia.

Eligible patients in the study signed the informed consent form the day before surgery. Patients followed standard preoperative fasting guidelines. Upon arrival in the operating room, standard monitoring included electrocardiography (ECG), pulse oximetry (SpO₂), and invasive arterial blood pressure monitoring via radial artery cannulation. Depth of anesthesia was monitored using the Bispectral Index (BIS), which was maintained between 40 and 60. Anesthesia was induced with sufentanil 0.4 μg/kg, propofol 2–2.5 mg/kg, and rocuronium 0.6 mg/kg. Intraoperative one-lung ventilation was maintained using a double-lumen endotracheal tube. Anesthesia was maintained using sevoflurane inhalation at 0.7–1.0 MAC, propofol infusion at 2–6 mg/kg/h, and remifentanil infusion at 0.1–0.4 μg/kg/min. Additional rocuronium (0.3 mg/kg) was administered as needed. During one-lung ventilation, tidal volume was maintained at 4–6 ml/kg with PEEP 5 cmH_2_O, and end-tidal carbon dioxide was maintained between 35–50 mmHg. Sevoflurane was discontinued when closing the surgical incision, with Propofol and Remifentanil dosages adjusted according to the BIS values. Intercostal nerve blocks were performed by surgeons using 0.5% ropivacaine. The research nurse then administered an intravenous loading dose of either Oliceridine 1.5 mg or Morphine 4 mg. Double-lung ventilation was restored after surgery completion. Following manual lung recruitment, patients were transferred to the Post-Anesthesia Care Unit (PACU) with the endotracheal tube.

### Postoperative management in PACU

Upon returning to PACU, Sugammadex was routinely administered to reverse neuromuscular blocking agents. The double-lumen endotracheal tube was removed upon meeting extubation criteria: complete consciousness; response to verbal commands; restored swallowing and cough reflexes; adequate muscle strength; tidal volume > 6 ml/kg; and respiratory rate ≥ 12 breaths/minute. Arterial blood gas analysis was performed within 30 min after extubation. During PACU stay, noninvasive ventilator (NIV) was initiated if SpO_2_ < 90%, arterial blood gas analysis showed PO_2_ < 60 mmHg, or PCO_2_ > 50 mmHg.

### Postoperative analgesia

NRS pain scores were assessed 5 min after extubation. Rescue analgesic therapy (Oliceridine 0.5 mg or Morphine 1 mg) was administered if NRS > 4. Pain scores were reassessed every 15 min, and rescue doses were repeated as needed until NRS < 3. After returning to the ward, patients received scheduled ketorolac tromethamine 30 mg twice daily and were encouraged to use oral nonsteroidal anti-inflammatory drugs (NSAIDs) for pain control. Pethidine was administered as rescue analgesia for uncontrolled pain.

### Outcome measures

The primary outcome was time to extubation, defined as the time from discontinuation of anesthetic agents to endotracheal tube removal, assessed and reported by PACU physicians according to standard criteria.

Secondary outcomes included the NRS score at 5 min post-extubation; Alertness recovery time was defined as the interval from cessation of anesthetics to achieve a Modified Observer's Assessment of Alertness/Sedation Scale (MOAA/S) score ≥ 4; Proportion of patients requiring rescue analgesia in the PACU and the median number of rescue doses per patient; Adverse events included respiratory depression (requiring noninvasive ventilatory support), agitation (Riker Sedation-Agitation Scale(SAS) score ≥ 5), and hypertension (mean arterial pressure > 20% above baseline). PACU length of stay was defined as the time from PACU admission to achieving an Aldrete score ≥ 9;Postoperative 24-h QoR-15 scores, incidence of 24-h postoperative nausea and vomiting; time to first oral intake (hours from surgery end), and time to first ambulation (hours from surgery end).

### Statistical analysis

Based on preliminary study data showing that extubation times in the oliceridine group was 13.9 ± 5.5 min compared with 21.9 ± 11.5 min in the morphine group. we calculated the sample size using PASS 11 software. To achieve a statistical power of 80% and a significance level of 0.05, we calculated that 80 patients (40 per group) were required in this trial. Accounting for possible dropouts, the final sample size was set at 100 patients (50 per group).

Data were analyzed using the SPSS 25.0 software package (IBM Corp., Armonk, NY, USA). Data distribution was assessed using the Shapiro–Wilk test. Data are reported as mean ± standard deviation (SD) or median (interquartile range, IQR) as appropriate. Categorical data are presented as counts (percentages). For continuous variables conforming to a normal distribution, independent-samples t-tests were employed to compare the two groups. For data not adhering to a normal distribution, the Mann–Whitney U tests (Wilcoxon rank-sum tests) was utilized. Categorical variables were compared using Chi-square tests or Fisher's exact tests. The confidence intervals for the mean difference and the median difference were derived from the t-test and the Hodges-Lehmann estimator (associated with the Mann–Whitney U test), respectively. A p-value < 0.05 was considered statistically significant.

## Results

**Fig. 1:**
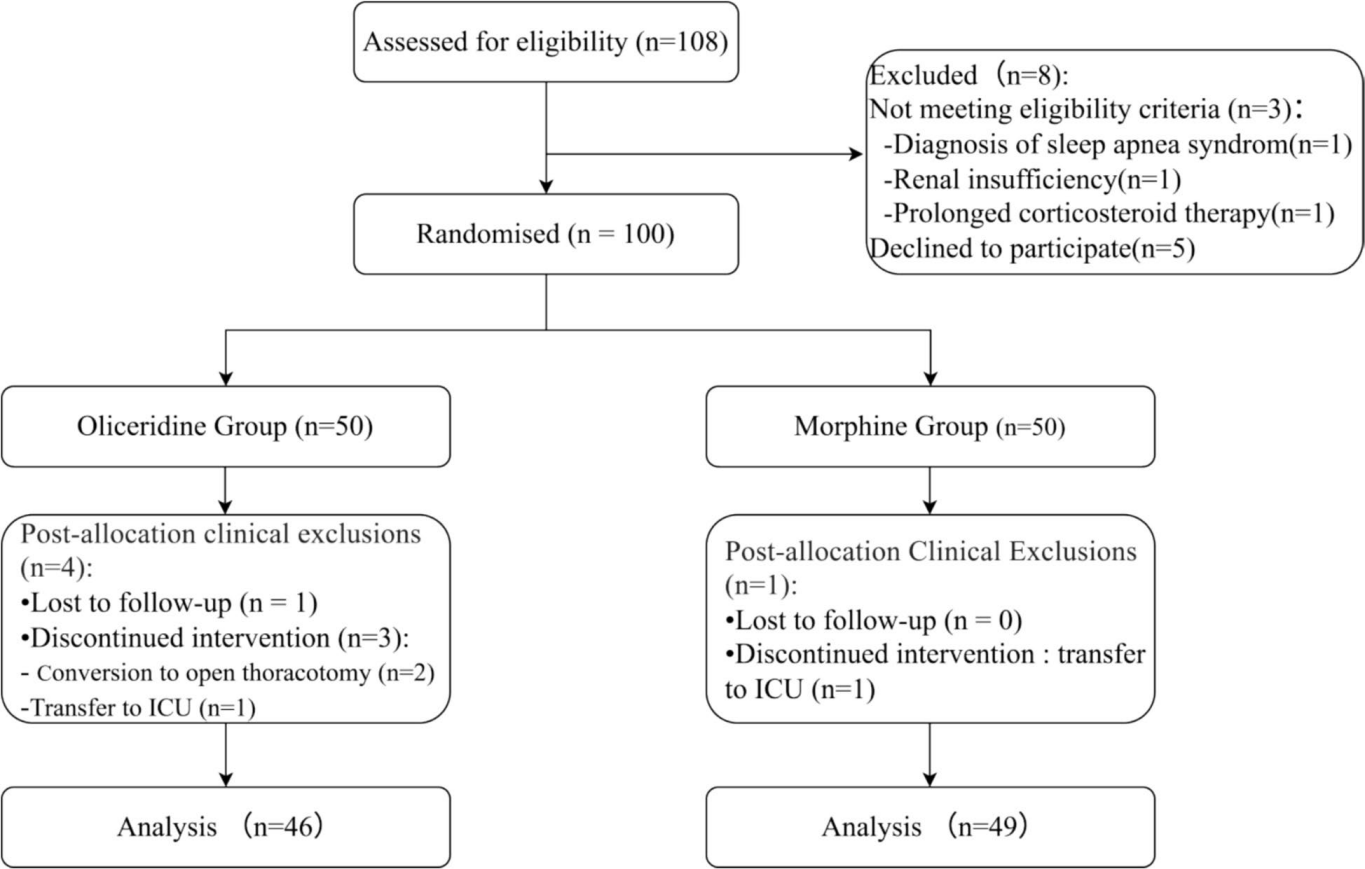
Trial flow diagram

A total of 108 patients were screened (Fig. 1). Of these, 8 patients were excluded, and 100 patients were randomized to the oliceridine group or the morphine group. In the Oliceridine group, 4 patients were excluded because 2 patients required conversion to open thoracotomy, 1 patient was transferred to the intensive care unit (ICU) because of bleeding, and 1 patient was lost to follow-up. A patient in the morphine group was excluded due to transfer to the ICU for intraoperative arrhythmia. Consequently, data from 95 patients (46 in the oliceridine group and 49 in the morphine group) were analyzed.

The baseline characteristics of both groups were well balanced (Table 1).

**Table 1:** Patient characteristics

	**Oliceridine (n = 46)**	**Morphine (n = 49)**
Age, years	52 ± 12	54 ± 12
Gender, female	32(70)	25(51)
Height, cm	160 ± 8	162 ± 8
Weight, kg	60 ± 9	61 ± 11
ASA class Ⅱ/Ⅲ	36/10	36/13
Operation time, min	113(82–150)	107 (80–162)
Anesthesia time, min	156(119–173)	166(118–195)
Sufentanil, μg	25 ± 4	25 ± 4
Remifentanil, mg	1.2(0.9–1.7)	1.2(1–1.7)
Sevoflurane cessation time, min	30 ± 11	29 ± 9
Surgery types,n (%)		
Lobectomy	9(19.6)	5(10.2)
Segmentectomy/Wedge resection	31(67.4)	38(77.6)
Mediastinal surgery	6(13.0)	6(12.2)

Subject and baseline characteristics. Data are presented as mean ± SD, median (IQR), or number (%). Continuous data are presented as mean ± SD were compared with t-test; Data presented as median (IQR) were compared using Wilcoxon rank-sum test; categorical data are presented as n (%) using Chi-square test

Compared with the morphine group, patients in the oliceridine group had a significantly shorter time to extubation (12 min vs 20 min; *P* < 0.001), a faster alertness recovery(18 min vs 29 min; *P* < 0.001), and a shorter PACU length of stay(65[60, 64]min vs 77[60, 83]min; *P* = 0.014). Postextubation NRS scores were lower in the oliceridine group (3 [3, 4]) than in the morphine group (5 [4, 5]; *P* < 0.001). The proportion of patients requiring rescue analgesic was lower in the oliceridine group than in the morphine group (45.7% vs 77.6%, *P* = 0.001), and the median number of rescue per patient was lower (1 [0, 1] vs 2 [1, 2]; *P* < 0.001); No statistically significant differences were observed between groups in the incidence of respiratory depression (10.9% vs 20.4%; *P* = 0.203), agitation (4.3% vs 12.2%; *P* = 0.310), or hypertension incidence (4.3% vs 14.3%; *P* = 0.193). However, the overall incidence of adverse events was higher in the morphine group than in the oliceridine group (38.8% vs 19.6%; *P* = 0.04) (Table 2).

**Table 2:** Outcomes During Postanesthesia Care Unit (PACU) Stay

	**Oliceridine** **(n = 46)**	**Morphine** **(n = 49)**	***P***	**Mean/Median Difference** **(95% CI)**
Time to Extubation, min	12.3 ± 4.6	19.9 ± 8.3	< 0.001	−7.6(−10.4–−4.9)
Alertness Recovery Time, min	18.5 ± 4.9	28.7 ± 9.8	< 0.001	−10.2(−13.3–−7.1)
NRS scores	3.0(3.0–4.0)	5.0(4.0–5.0)	< 0.001	−1.0(−2.0–−1.0)
Number of Rescue per Patient	0.0(0.0–1.0)	2.0(1.0- 2.0)	< 0.001	−1.0(−1.0–−1.0)
Patients Requiring Pain Rescue	21(45.7)	38(77.6)	0.001	
PaCO_2,_mmHg	42.3(38.0–46.2)	43.0(41.4–47.0)	0.106	−1.7(−4.0–0.3)
PaO_2_, mmHg	137.0(105.9–224.8)	134.8(96.3–168.1)	0.461	9.2(−13.8–35.0)
Length of PACU Stay, min	60.0(60.0- 63.8)	60.0(60.0–82.5)	0.014	0.0(−10.0–0.0)
Adverse Events	9(19.6)	19(38.8)	0.04	
Agitation	2 (4.3)	6 (12.2)	0.310	
Hypertension	2 (4.3)	7 (14.3)	0.193	
Respiratory Inhibition	5(10.9)	10(20.4)	0.203	

Data are presented as mean ± SD, median (IQR), or n (%). NRS, Numerical Rating Scale (0–10). PACU, Post-Anesthesia Care Unit. The Fisher exact test was used for categorical variables with low cell counts

After returning to the ward, there were no statistically significant differences between the two groups in any assessed 24-h postoperative recovery metrics, including QoR-15 scores, time to first oral intake, time to first ambulation, and gastrointestinal adverse events (*P* > 0.05) (Table 3).

**Table 3:** Postoperative Outcomes at 24 Hours

	**Oliceridine** **(n = 46)**	**Morphine** **(n = 49)**	***P***	**Mean/Median Difference** **(95% CI)**
QoR-15 Score	128.0(116.0–133.0)	124.0(117.0–132.5)	0.404	2.0(−3.0–6.0)
Time to First Oral Intake,h	7.5(6.4–9.1)	8.0(7.3–9.0)	0.075	−0.5(−1.5–0.0)
Time to First Ambulation, h	21.8 ± 3.4	23.0 ± 3.4	0.096	−1.2(−2.6–0.2)
PONV	8(17.4)	15(30.6)	0.133	
Vomit	2(4.3)	7(14.3)	0.193	
Nausea	6(13.0)	8(16.3)	0.652	

Data are presented as median (IQR), mean ± SD, or n (%). QoR-15, Quality of Recovery-15 questionnaire; PONV, Postoperative nausea and vomiting. The Fisher exact test was used for categorical variables with low cell counts

## Discussion

This randomized controlled trial demonstrates that compared to morphine, oliceridine significantly enhances the quality of early recovery in patients undergoing VATS. Specifically, oliceridine use was associated with faster time to extubation, quicker return of alertness, superior immediate postoperative pain control, fewer adverse events in the PACU, and a shorter PACU stay.

Studies suggest that improving early recovery quality can facilitate the recovery of patients’ physiological functions, reduce postoperative complication rates and decrease length of stay [8, 9]. Time to extubation was selected as the primary outcome for assessing early recovery quality because it is an objective and clinically relevant metric reflecting early recovery of respiratory function and readiness for extubation, which aligns with ERATS goals of minimizing ventilator time and promoting rapid postoperative recovery [1]. Oliceridine, a G protein-biased μ-opioid receptor agonist, may offer a potential advantage in this context. Its mechanism of action involves selective activation of the G protein signaling pathway for potent analgesia, while minimizing β-arrestin recruitment, a pathway associated with many common opioid-related adverse effects [10, 11]. Consistent with this mechanistic profile, clinical studies have reported that oliceridine, compared to morphine, exhibits lower incidences of gastrointestinal adverse reactions, respiratory depression, dizziness, drowsiness, sedation, and delirium [7, 11–13]. Based on these favorable safety and tolerability characteristics**,** we hypothesized that oliceridine-based postoperative analgesia would support, rather than delay, patient emergence and extubation. Our findings confirm this hypothesis: patients administered oliceridine achieved earlier extubation (by 40%), faster recovery of alertness (by 38%), and met PACU discharge criteria more rapidly compared to those receiving morphine.

Postoperative respiratory depression poses a significant risk, potentially leading to hypoxia or hypercapnia in patients. Patients undergoing thoracic surgery have reduced compensatory capacity for ventilatory dysfunction due to diminished lung volume and predisposition to postoperative atelectasis. Severe cases may necessitate reintubation. Thus, ensuring respiratory safety alongside effective analgesia is paramount in thoracic surgery. Although oliceridine is associated with a more favorable respiratory safety profile with lower incidences of hypoxemia and hypercapnia compared to morphine [14–17], our study did not demonstrate statistically significant differences in PaO₂ (*P* = 0.461), PaCO₂ (*P* = 0.106), or the incidence of respiratory depression requiring intervention (10.9% vs. 20.4%, *P* = 0.203). However, the observed numerical reduction in respiratory depression (46.6% relative reduction) warrants further investigation to confirm potential respiratory safety benefits.

To mitigate opioid-related side effects, ERATS emphasizes multimodal analgesia, shifting away from opioid-centric regimens towards combining regional techniques with pharmacotherapy for optimal pain control with minimal adverse events [18–21]. Evidence supports a "restricted opioid" strategy within multimodal analgesia (i.e., short-term, low-dose, as-needed opioids) as beneficial over "opioid-free" approaches, especially for moderate-to-severe acute pain [4, 20, 22]. Thoracic surgery, whether employing open or minimally invasive techniques, is frequently associated with severe postoperative pain. Inadequate pain control hinders early mobilization and increases the risk of chronic postsurgical pain, negatively impacting quality of life [18, 23]. Adhering to ERATS principles, we employed intercostal nerve blockade combined with short-acting opioid supplementation (oliceridine or morphine) intraoperatively and in PACU. Postoperatively, both groups received scheduled NSAIDs (ketorolac) with pethidine administered as rescue medication for breakthrough severe pain on the ward. Based on prior Phase III trials (APOLLO-1 and APOLLO-2), using recommended loading (oliceridine 1.5 mg vs morphine 4 mg) and rescue doses (oliceridine 0.5 mg vs morphine 1 mg) [6, 7], oliceridine provided superior analgesia in the immediate postoperative period, evidenced by lower NRS scores and reduced rescue analgesia requirements. However, the lack of difference in pethidine use on the ward suggests the analgesic advantage of short-term oliceridine administration does not extend beyond the PACU phase. It should be noted that the doses of Intervention drugs were based on prior clinical trials and common clinical practice, but may not reflect a fully equianalgesic ratio. This dosing strategy could have contributed to the observed differences in early pain scores and rescue analgesia requirements in the PACU. Therefore, our findings regarding superior immediate analgesia with oliceridine should be interpreted within this context, and future studies employing strictly equianalgesic rescue regimens are warranted to confirm these results.

Postoperative nausea and vomiting (PONV) is a common adverse event impacting patients' recovery quality. While thoracic surgery itself is not a primary PONV risk factor, known contributors include female gender, volatile anesthetics, opioids, and prolonged anesthesia/surgery time [1]. Reported PONV rates after thoracic surgery range from 16.9% to 31.7% [24, 25], influenced by assessment methods, analgesic regimens, and patient factors like gender distribution. While some studies have reported lower rates of postoperative nausea and vomiting (PONV) with oliceridine compared to morphine [6, 17], the incidence of PONV within 24 h in this study did not differ significantly between groups (17.4% in the oliceridine group vs. 30.6% in the morphine group; *P* = 0.133). Among patients who experienced gastrointestinal adverse events, vomiting was reported in a larger proportion of patients in the morphine group (47%) than in the oliceridine group (25%), although this difference was not statistically significant. It should be noted that the oliceridine group had a higher proportion of female patients (69.6% vs. 51.0%), which is a known risk factor for PONV. To account for this potential confounding effect, we performed a logistic regression analysis on the incidence of PONV. After adjusting for sex, there was also no statistically significant difference in PONV between the oliceridine and morphine groups (OR = 0.413, 95% CI 0.15–1.13, *P* = 0.085).

The absence of a significant difference in the time to first oral intake and ambulation may be attributed to the non-standardized postoperative management and the homogeneous analgesic regimen used beyond the PACU phase, which superseded the short-term intraoperative intervention. Studies suggest that early ambulation, within 1 h post-extubation, may effectively reduce length of stay without increasing complications [26, 27]. Effective analgesia and minimal side effects are prerequisites for such mobilization. This appears to be a potential advantage of oliceridine, warranting further investigation in future studies.

Given that the study intervention was administered exclusively during PACU stay, we selected the 24-h QoR-15 as the sole longer-term endpoint. The results demonstrated no significant between-group difference in recovery quality scores at 24 h, indicating that its short-term analgesic superiority did not yield sustained clinical benefits at 24 h. This attenuation of long-term differences may be attributable to the standardized multimodal analgesic regimen implemented after PACU discharge, which effectively equalized recovery trajectories between the two groups.

These findings have important implications for clinical practice and future research. First, while oliceridine may promote faster early recovery—a key objective of enhanced recovery pathways—its benefits appear to be confined to the period of active drug administration. Second, given the substantially higher acquisition cost of oliceridine compared to morphine in China (in our region, the price of oliceridine is approximately USD 5.79 per 2 mL:2 mg ampoule, while morphine is approximately USD 0.53 per 1 mL:10 mg ampoule), its transient clinical advantages must be carefully weighed against economic considerations. Therefore, to determine its practical value in routine clinical use, further studies are warranted to investigate whether extending oliceridine therapy into the ward phase can sustain recovery benefits and justify the associated costs, supported by longer follow-up and formal cost-effectiveness analyses.

In summary, the findings from this study demonstrate that oliceridine, when used for emergence analgesia after VATS, is associated with several advantages in the early postoperative phase compared to traditional morphine. These include facilitating faster recovery of respiratory function and consciousness, as well as providing more effective initial pain management. This profile warrants its consideration within enhanced recovery protocols.

### Limitations

Our study has several limitations. The sample size was determined by the primary outcome (extubation time), meaning the study might be underpowered for certain secondary endpoints. The pharmacologic intervention was also confined to the perioperative period in the PACU, and because postoperative analgesia was standardized, we could not assess the long-term effects of the study drugs. To evaluate sustained outcomes, future studies would need to extend the drug intervention period. Additionally, the rescue dosing regimen, though aligned with previous clinical trials, might not be fully equianalgesic, which could have affected the results regarding early analgesic requirements. It is also important to note that extubation time, while objective, is not a comprehensive measure of recovery quality. Lastly, the single-center design may affect the generalizability of our results, warranting confirmation through multicenter trials.

## Conclusion

In conclusion, compared with morphine, short-term administration of oliceridine for emergence analgesia following VATS significantly enhances the quality of immediate recovery in the PACU. The benefits encompass faster extubation, quicker return of alertness, superior early postoperative pain control with reduced rescue analgesia requirement, and a lower overall incidence of adverse events. These results establish oliceridine as a valuable analgesic option in ERAS pathways for VATS, particularly for achieving early recovery milestones. However, the benefits appear confined to the peri-extubation period, necessitating further research to evaluate its long-term outcomes, safety beyond the PACU, and cost-effectiveness.

## Data Availability

De-identified individual participant data and the study protocol are available from the corresponding author upon reasonable request.

## Abbreviations

VATS: Video-assisted thoracoscopic surgery
PACU: Post-anesthesia care unit
NRS: Numerical Rating Scale pain scores
QoR-15: Quality of Recovery-15 scores
ERATS: Enhanced Recovery After Thoracic Surgery
ERAS: Enhanced Recovery After Surgery
BMI: A body mass index
ECG: Electrocardiography
SpO_2_: Pulse oximetry
BIS: Bispectral Index
NIV: Noninvasive ventilator
NSAIDs: Nonsteroidal anti-inflammatory drugs
MOAA/S: Modified Observer's Assessment of Alertness/Sedation Scale score
SAS: Riker Sedation-Agitation Scale Score
SD: Standard deviation
IQR: Interquartile range
ICU: Intensive care unit
PONV: Postoperative nausea and vomiting
